# Profile and obstetric outcome of teenage pregnancies compared with pregnant adults at a district hospital in KwaZulu-Natal

**DOI:** 10.4102/safp.v63i1.5290

**Published:** 2021-09-07

**Authors:** Olaolu I. Ogunwale, Selvandran Rangiah

**Affiliations:** 1 Department of Family Medicine, Faculty of Health Sciences, University of KwaZulu-Natal, Durban, South Africa

**Keywords:** teenage pregnancy, profile, outcomes, obstetric risk, district hospital

## Abstract

**Background:**

Teenage pregnancy remains a major public health concern and a challenge for developing countries. Young maternal age can lead to serious physical, social and psychological consequences as teenage mothers are less likely to gain full educational potential and are at higher risk of poverty and complications of pregnancy. The objective of the study was to describe the profile and obstetric outcome of teenage pregnancy compared with that of pregnant adults at a district hospital in KwaZulu-Natal.

**Methods:**

A retrospective descriptive study utilising data obtained from randomly selected hospital records of 216 teenage mothers compared the socio-demographic profile, foetal and maternal outcomes to that of pregnant adults.

**Results:**

The mean age of the teenage group was 17.6 and 26.0 years for the adults (control group). Both groups had a remarkable booking status (97.2% vs. 100%) and antenatal attendance (62.5% vs. 66.2% with ≥ 5 visits). No significant difference in anaemia, caesarean delivery and obstetric complications were found in both groups. There was, however, a significant risk of hypertensive disorder of pregnancy (39.8% vs. 26.4%, *p* = 0.030) and higher risk of episiotomy being carried out during delivery (31.5% vs. 13.0%). On the other hand, the control group had a significant higher risk of HIV infection (12.5% vs. 38.4% *p* = 0.000).

**Conclusion:**

The study showed that teenage pregnancy has a similar obstetric risk to adult pregnant patients except for hypertension disorder of pregnancy. Although this study demonstrated improved antenatal attendance by pregnant teenagers, the psychosocial impact on young mothers requires further research.

## Introduction

Teenage pregnancy is defined as pregnancy that occurs in women aged between 10 and 19 years, with some authors distinguishing teenagers aged between 15 and 19 years from younger teenagers aged between 10 and 14 years.^1^ Annually, an estimated 21 million girls aged 15–19 years in developing countries become pregnant and approximately 12 million of them give birth, whilst almost 777 000 births occur in adolescent girls younger than 15 years.^2^

In both developing and developed countries, teenage pregnancy remains a social problem that is more prevalent in marginalised communities and driven by poverty, inadequate education and scarce employment opportunities.^2,3,4^

Teenage pregnancy, a major public health issue for both mother and baby, is often associated with adverse health outcomes.^5,6^ Pregnant teenagers are more prone to developing pregnancy-related complications such as obstructed labour, obstructed fistulae, preterm delivery small for age babies, developmental abnormalities and even maternal death.^7,8^ Complications resulting from pregnancy and delivery are the major causative factor of death occurring amongst women whose ages are between 15 and 19 years in the developing countries.^9,10,11^

There are biological, psychological, socio-demographic and behavioural factors that may influence the outcome of a teenage pregnancy.^12,13^ These include, lack of information and access to family planning services, single teenagers, lack of formal education, low socio-economic status, gender-based violence, substance use and stigmatisation by the community.^14,15,16,17,18,19^

In South Africa, teenage pregnancy is common with a prevalence of 47 births per 1000 girls aged 15–19 per annum.^20^ The international and local literature has focused on causes and contributing factors.^10^ There is however a paucity of data on obstetric outcomes in teenage pregnancy in South Africa. The few studies conducted on teenage pregnancy in Africa has revealed inconsistent and inconclusive findings.^20^ The aim of this study was to determine the profile and obstetric outcome of pregnancy in teenagers compared with pregnant adults at a district hospital in KwaZulu-Natal (KZN).

## Material and method

This study was an observational descriptive study carried out at Lower Umfolozi Memorial hospital (LUDWMH), Empangeni, KZN between January 2017 and June 2017.

The profile and obstetric outcome of 216 pregnant teenage women who were admitted and delivered at LUDWMH was analysed. The data were obtained from their maternal records, which were systematically selected and the results were analysed and compared with an equal number of maternal records of adult pregnant women. Systematic sampling was adopted using every third record after the first patient record was selected. Selection of every third file was based on our previous estimate of deliveries and this provided the sample size required to provide a statistical power of 80%. Women who delivered either at home, private hospital or other facilities belonging to non-governmental organisations were excluded from the study. The reason for this exclusion was that these records were not available to the researcher.

The socio-demographic characteristic variables of the two groups of women, that is, the pregnant teenagers and the pregnant adults, complications of pregnancy and delivery and neonatal outcome were extracted.

Data were entered on a Microsoft Excel spreadsheet, coded and then exported to Statistical Package for Social Sciences (SPSS) version 25 software for statistical analysis. Descriptive statistical analyses of the data (frequencies and percentages) are presented. Proportions were compared using the chi-squared test. Where appropriate, a *p* < 0.05 was considered statistically significant.

### Ethical considerations

Ethical approval was obtained from the University of KwaZulu-Natal Biomedical Research Ethics Committee (reference BE202/15) and the KZN Department of Health and the management of the hospital where the study was conducted.

## Results

The mean ages of the participants were 17.6 and 26.0 for the teenagers and adults, respectively. A high number of singles was found amongst the teenagers (215, 99.5%) whilst most of the adults had more than one child (141, 65.3%). The booking trend in both groups was similar (teenagers: 210, 97.2% vs. adults: 216, 100.0%) with more than five antenatal visits done by 62.5% of the teenagers and 66.2% of the adults. The positive HIV status was higher in the adult group (83, 38.4%) than in the teenage group (27, 12.5%) (Table 1).

**TABLE 1:** Demographic data.

Variable	Study group (teenagers) (*n* = 216)			Control group (adults) (*n* = 216)			*p*
	Mean	*n*	%	Mean	*n*	%	
**Maternal age**	17.6 ± 1.32	-	-	26.0 ± 4.46	-	-	0.000
**Marital status**							
Married	-	1	0.5	-	11	5.1	0.003
Single	-	215	99.5	-	205	94.9	-
**Parity**							
Primiparous	-	199	92.1	-	75	34.7	0.000
Multiparous	-	17	7.9	-	141	65.3	-
**Booking status**							
Booked	-	210	97.2	-	216	100.0	0.006
Unbooked	-	6	2.8	-	0	0.0	-
**Antenatal attendance**							
0–4 visits	-	81	37.5	-	73	33.8	0.002
≥ 5 visits	-	135	62.5	-	143	66.2	-
**HIV status**							
Seropositive	-	27	12.5	-	83	38.4	0.000
Seropositive on treatment	-	27	100.0	-	81	97.5	-
**Syphilis status**							
Seropositive	-	2	0.9	-	3	1.4	0.654
**Rhesus group**							
Negative	-	4	1.9	-	1	0.5	0.178
Positive	-	12	98.1	-	215	99.5	-

The pregnancy-related complications for both groups are represented in Table 2. Hypertensive disorders were significantly higher in the teenage group (86, 39.8%) compared with the adult group (57, 26.4%), *p* < 0.05. Teenagers had a lower percentage of anaemia (39.8% vs. 43.1%) but a higher percentage of proteinuria when compared with the adults (28.2 % vs. 25.5%).

**TABLE 2:** Pregnancy-related complications.

Variable	Study group (teenagers) (*n* = 216)		Control group (adults) (*n* = 216)		*p*
	*n*	%	*n*	%	
Anaemia in pregnancy	86	39.8	93	43.1	0.491
Hypertensive disorders	86	39.8	57	26.4	0.003
Proteinuria (1+ or more)	61	28.2	55	25.5	0.516
Eclampsia	8	3.7	2	0.9	0.055
Abruptio placenta	3	1.4	1	0.5	0.316
Placenta previa	1	0.5	1	0.5	1.000
Preterm labour	31	14.4	35	16.2	0.079
Prolong rupture of membrane (> 24 h)	9	4.2	3	1.4	0.059

The pregnancy outcomes are tabulated in Table 3 for both groups of pregnant women. Teenagers and adults had majority singleton deliveries (214, 99.1% vs. 210, 97.2%) with 75.5% of the former and 74.5% of the latter delivering at term. Both groups had similar preterm delivery rates (50, 23.1% vs. 48, 2.2%). There was no significant difference for induction and augmentation of labour in the two groups. Caesarean section rates were higher in the adult group (117, 54.2% vs. 99, 45.8%), whilst birthweight above 2.5 kg was similar in the teenagers and adults. Whilst the number of still births was low, birth asphyxia occurred in 23 (10.6%) teenagers and 19 (8.8%) adults. More episiotomies were performed in the teenagers (68, 31.5%) with first degree perineal tears occurring in 22 (10.5%) teenagers and 25 (11.6%) adults.

**TABLE 3:** Outcome of pregnancy.

Variable	Study group (teenagers) (*n* = 216)		Control group (adults) (*n* = 216)		*p*
	*n*	%	*n*	%	
**Number of foetuses**					
Singleton	214	99.1	210	97.2	0.154
Twin	2	0.9	6	2.8	-
**Duration of pregnancy**					
Pre-term	50	23.1	48	22.2	0.438
Term	163	75.5	161	74.5	-
Post-term	3	1.4	7	3.2	-
**Induction of labour**	29	13.4	29	13.4	1.000
**Augmentation of labour**	16	7.4	18	8.3	0.858
**Mode of delivery**					
Caesarean section	99	45.8	117	54.2	0.225
Normal vertex delivery	115	53.2	97	44.9	-
Forceps	1	0.5	0	0.0	-
Vacuum	1	0.5	2	0.9	-
**Birthweight**					
1 kg or less	2	0.9	7	3.2	0.080
1.1 kg – 2.50 kg	53	24.5	43	19.9	-
2.51 kg – 3.50 kg	136	63.0	116	53.7	-
> 3.50 kg	25	11.6	50	23.1	-
**Still birth**					
FSB	3	1.4	0	0.0	0.100
MSB	3	1.4	7	3.2	-
**Birth asphyxia**	23	10.6	19	8.8	0.517
**Apgar score < 4**	10	4.7	7	3.2	-
**Perineal tear**					
1st degree	22	10.7	25	11.6	0.685
2nd degree	2	0.9	5	2.3	-
3rd degree	1	0.5	2	0.2	-
**Episiotomy**	68	31.5	28	13.0	0.000
**Maternal death**	1	0.5	0	0.0	0.100

FSB, fresh still birth; MSB, macerated still birth.

## Discussion

In this study, most teenagers (215, 99.5%) were not married, as presented in Table 1. This finding is consistent with the South African Census 2016 that states 95.1% of teenagers in the country are unmarried and a document published through the Department of basic education in 2009 supporting the fact that marriage amongst teenagers is less common and usually occurs late in their reproductive life.^21^ This study’s outcome seems to be inconsistent with what is highlighted in developing countries where child marriage is common and has been associated with teenage pregnancy.^22^

Although more teenagers presented with primigravid pregnancies, both adults and teenagers had a similar booking trend and attendance of more than five antenatal visits. This finding differs from other studies, which suggest that stigma and embarrassment prevent teenagers from attending antenatal visits.^23,24^ The cost of transport, limited knowledge of antenatal care programmes and fear of HIV testing were previously argued for poor antenatal visits amongst teenagers.^24^ This was not evident in this study. Fulpagare et al.,^25^ on the other hand have demonstrated better antenatal attendance in teenagers compared with adults.

Contrary to the study of Christofides et al.,^26^ this study did not demonstrate a high risk of HIV infection amongst the pregnant teenagers of which 12.5% were seropositive for HIV infection compared with 38.4% in the adult group (*p* = 0.000). The risk of HIV infection, however, increases with age and parity supported by the data of other studies.^26,27^

Hypertensive disorders were significantly higher amongst pregnant teenagers compared with the pregnant adults, a finding that is consistent with several studies.^28,29,30^ Early detection of maternal complications is central to preventing adverse maternal and foetal complications. Although antenatal attendance in this study was good, hypertension in pregnancy occurred till delivery suggesting a pathophysiological basis for this condition in the light of good pharmacological intervention from the early stage of gestation. A study in Thailand, however, linked poor antenatal care to increased rate of pregnancy-induced hypertension and adverse foetal outcomes.^31^

Pregnant teenagers living in communities in the Empangeni subdistrict of KZN have similar obstetric outcomes to that of pregnant adult patients corroborating the findings of Hoque’s^32^ study in the same community. Teenagers did not have an additional risk of low birthweight, preterm deliveries and still births.

However, in this study, 39.8% of teenagers did have anaemia in pregnancy, 39.8% had hypertensive disorders, 28.2% had proteinuria and 14.4% presented with preterm labour. These findings are similar to that of several studies indicating that teenagers are at a high risk of pregnancy-related complications.^33,34,35^

On the outcome of pregnancy, this study showed few significant differences between pregnant teenagers and pregnant adults. Most teenagers (75.5%) delivered at term with the mode of delivery being mainly normal vaginal delivery. The caesarean section rate in this study was 45.8% for the teenagers compared with 54.2% in the adults, despite some authors claiming that teenagers are unlikely to deliver via caesarean section.^36^ Although there was no significant difference in both groups, caesarean section has a high risk of obstetric complications with subsequent deliveries likely to follow the previous mode.^37^

In this study, 74.6% of teenagers delivered babies with a birthweight above 2.5 kg despite reports of very low birthweights in the literature.^38,39,40^ This was a similar finding to the adult group of which 76.8% delivered babies with a birthweight above 2.5 kg.

Previous studies showed an increase in preterm delivery, low birthweight and small for gestational age babies, which is in contrast to findings from our study.^34,35^

Although the literature on teenage pregnancy is controversial, there is agreement that teenage pregnancies have a high risk of developing obstetric complications. In this study, the obstetric risk in pregnant teenagers was similar to that of the pregnant adults with the exception of hypertensive disorders. However, the absolute rate of anaemia in pregnancy (39.8%), proteinuria (28.2%), the caesarean section rate (45.8%) and preterm labour (14.4%) amongst teenagers is of concern, despite early and frequent antenatal care. Further studies exploring the social impact of teenage pregnancies are recommended.

Healthcare provider awareness, focused and prompt care may prevent the development of complications.

## Limitation of study

This study was a retrospective chart review; hence, this could be a limitation of the study as information may be inadequately recorded by service providers or totally omitted. Information might be provided in the records about services not rendered resulting in false measurement. Furthermore, psychosocial factors were not routinely recorded in the charts.

## Conclusion

Although this study demonstrated a similar obstetric risk in pregnant teenagers and pregnant adults, teenage pregnancy remains a major public health concern. The absolute rates of anaemia, hypertension in pregnancy and proteinuria amongst teenagers was high. However, contrary to previous evidence of poor antenatal attendance by pregnant teenagers, the antenatal attendance of pregnant teenagers in this study was remarkable. Healthy birthweights, low incidence of still births, birth asphyxia and low Apgar scores may be associated with good antenatal attendance and good obstetric care.

Appropriate antenatal and postnatal care are fundamental in reducing perinatal and maternal morbidity and mortality. The continuous evaluation of data in different geographical regions will help to improve strategies in the care of pregnant teenagers. Health and sex education is recommended to improve accessibility to family planning and safeguard the reproductive health of teenagers.
